# Some Practical Hints upon the Management of Chronic Cases

**Published:** 1889-08

**Authors:** 


### HOMŒOPATHIC PHYSICIAN,

A MONTHLY JOURNAL OF

HOMŒOPATHIC MATERIA MEDICA AND CLINICAL MEDICINE.

If our school ever give up the strict inductive method of Hahnemann, we are lost, and deserve only to be mentioned as a caricature in the history of medicine."—CONSTANTINE HERING.

Vol. IX. AUGUST, 1889. No. 8.

### SOME PRACTICAL HINTS UPON THE MANAGEMENT OF CHRONIC CASES.

[Extracted from proceedings I. H. A.]

At the recent meeting of the I. H. A., a paper, written by Dr. Wm. P. Wesselhœft, was read, entitled, "Practical Hints in the Management of Chronic Cases." The paper was full of just such practical hints as one would expect from one of Dr. Wesselhœft's medical experience and homœopathic knowledge. He especially recommended that any prescription should be given ample time to act that caused an improvement in the patient's mental condition, also when the patient seemed to feel more comfortable, even though, in either case, the physical signs as yet showed no evidence of improvement. The discussion which followed the reading of Dr. Wesselhœft's paper is so instructive that we give it in full.

Dr. Butler—There are one or two points I desire to bring out; and in order to do so I will relate the case of an old gentleman of most excellent habits, but whose ancestors had probably not been. A careful examination of his case revealed a history of gout; he presented a perfect picture of the lithæmic diathesis; he had been under the treatment of a noted homœopathic physician, more noted as a physician than as a homœopath, and under his treatment had received various internal and external applications, and so forth. On coming under my care he complained of difficult locomotion. I thought (being seventy-four years of age) it arose from the heart; that in all probability it was the weakness of old age. But careful examination showed there was actual lack of co-ordination; further examination showed lack of co-ordination; the left patellar reflex absent, the right one much weakened. Closing the eyes showed that he immediately staggered, and without aid would have fallen. The symptoms indicated Belladonna. There were plenty of Belladonna symptoms and he got Belladonna. The remark that was made in the paper, "I feel better but I don't know whether I only seem to be better, but I feel better," was verified here. I could not detect the slightest improvement in his *physical condition*, but his mental condition was decidedly better. The single dose, in a high potency, and nothing more for a month; "and then he had a dandy attack of gout," though he had not had gout for a number of years. Then I ascertained that he had had symptoms of posterior spinal schlerosis after the first attack of gout. The attack of gout was exceedingly severe, and the indications were for Bryonia. What should I have done? The man was suffering severely; he was suffering in many ways, because the disease was of a virulent type; all the indications were for Bryonia. Was it my duty to wait for this attack of gout to recover spontaneously, or under the influence of Belladonna, or was I to relieve him of his present condition?

Dr. Bell—Wait; I don't know; wait.

Dr. Butler—I gave him a dose of Bryonia, and to my delight the gout disappeared as if by magic; and the peculiar symptoms (lack of co-ordination with loss of tendo reflex), when he got better, which he did very fast, vanished; there was improvement in his gait and return of the knee-jerk upon the left knee, and reflex action was considerably improved. It is too early to say what may be the result, but at this time he is decidedly better, and has improved in his power of locomotion. That is the point I wish to bring out; when the old conditions reappear, whether it is better to prescribe for them, *or wait*. It is a difficult question to decide, and one which it is *then* necessary to decide for the patient's benefit.

Dr. Long—Did the patient say he felt better?

Dr. Butler—During the attack of gout he did not complain of feeling better.

Dr. Long—Was his mental condition and physical condition improved?

Dr. Butler—Yes, sir.

President—It is an interesting subject. I hope you will keep it up.

Dr. H. C. Allen—I would like to know how many physicians have seen disease of the heart result from rheumatic affection; whether we have retrocessions involving the heart. I think I have never seen a case.

Dr. W. J. H. Emory—If any one would give an answer to Dr. Butler's question I should like to hear it.

Dr. H. C. Allen—I withdraw mine.

Dr. J. T. Kent—I have done just as Dr. Butler did a number of times; once in awhile it seemed to be acceptable, but as a rule it is a failure, so I wait. I have partly succeeded for a time, but the rule has been it is a failure to give medicine at the time he gave Bryonia. I am afraid to do it, for it complicates the case, and there is the mistake that you often lose your reckoning, because nobody knows so much about the patient as the doctor that had him in charge. Dr. Butler had that case, I had not.

Dr. Bell—I believe I would have treated the case like Dr. Butler, and for the life of me I cannot see why not. We cannot indulge the suffering of pain as severe as it was in this case; and I think that Dr. Butler did right. I think it is a dangerous procedure to wait—it is dangerous ground. Treat pain while we must, taking into consideration the conditions there are, I think we are justifiable in using any remedy. I think the remedy for his case is still acting. I don't think it is deflected by the action of Bryonia. Now, as to the use of a repertory; this is the paper I was wanting. It had my commendation. I think it is a most excellent paper; and the law of Homœopathy is that we are not simply following in the course laid down by Hahnemann. Use your repertory. I never think of going to see a patient without I have my repertory in my satchel. I used, in 1863, to carry a phlebotomy case. I was called to see a case of pleuro-pneumonia in a man who was of very plethoric constitution. He was suffering intensely, and lived three miles away in the country. I got within a mile of the house when I found I had forgotten my phlebotomy instruments. I went back and got them, and the first thing I did was to draw from that man half a gallon of blood—this was the first case I ever treated. I am the same way now with my repertory. Prejudice it may be, but however simple the case you must have your repertory. And it is the way you use your repertory that tells. If you show ignorance you will fail. I went one hundred and thirty-five miles into the country the other day into a neighborhood where they did not know what a homœopath was. The first thing I did was to take my repertory, lay it on the table, and the next thing was to take my medicine-case. I saw a symptom or two I did not know, and I reached for my repertory and investigated my case, and gave a good prescription. So we are not justifiable in leaving our repertories at home. You may just as well leave your medicines.

Dr. J. V. Allen—I am going back to Dr. Butler's case. I

think I remember that Dr. Lippe told me once in regard to Apis: "We have now a proving of Apis." Before we had Apis to cure an Apis case he said he would sometimes go along with Sulphur for awhile, then for awhile with Rhus tox., and so on. It took three remedies to cure a case which is now cured by Apis alone. He said each remedy would rid the patient of certain symptoms, and another would have to be given. Dr. Hering, in his *Materia Medica,* mentions several complementary remedies. Belladonna will go so far and Calc. carb. will have to take it up and finish the case.

Dr. Hitchcock—I would like to ask Dr. Butler had this patient been under other homœopathic treatment?

Dr. Butler—No, sir; the kind of homœopathic treatment he had the less said about it the better. He got Colchicum because he could not rest, if that is what you call homœopathic treatment.

Dr. Hitchcock—The point I wish to make is that the Belladonna which was given had counteracted the condition which had been brought about by the use of various remedies, and so the remedy which was curative, Bryonia cleared up the case.

Dr. Biegler—I am not prepared to say a great deal. The point that Dr. Butler makes out appears to me to be one of judgment—in fact, one of study and experience on the part of the physician. However, I do make the rule of not interfering with the remedy that has cleared up the mental condition when I find the remedy has made the patient feel better, and he knows not why, but the physical state remains. I never interfere. I don't remember if I have done so of late. It may be a long battle but it will be a successful one. The success will be assured by the perseverance and by adhering to the passive policy of not treating that case ( *i. e.,* with Bryonia). Well, a case may occur, such as Dr. Butler's, where, possibly, Bryonia may have been the remedy, and you may not be prepared to express an opinion whether the doctor was right or wrong in his proceedings. But, I do wish to declare that the rule of not interfering, that the observance by making it a rule in practice of not interfering when the patient expresses himself to be better —I do wish to express that as being the true policy to adhere to.

Dr. Long—This is a very interesting topic to me, and I certainly appreciate it, only it is impossible to bring an intelligent view before another physician of your own patient, a familiar use of your judgment, or expression of your judgment. I should like to ask one question. How long did Dr. Butler wait?

Dr. Butler—Three days.

Dr. Long—We had the same question in our club in Philadelphia a short time ago in regard to the totality of the symptoms, and each physician described what we mean by the totality of the symptoms. Is it the whole duration of the sickness from the very beginning of visiting your case back to the whole history of the case; or is it only the expression of the chronic condition, just as it takes in all the sickness a man has ever had or most probably had; or is it the acute attack of the chronic condition? Dr. Butler prescribed for each attack of the chronic condition. We are unable to say how much of a cure is here accomplished—time alone will tell. Had the patient's mental condition improved and he yet had his physical ailment? If he had, Bryonia has done good.

Dr. Bell—I feel confident that Dr. Butler acted right; both mental and physical symptoms were changed, and in accordance with that change he gave Bryonia.

Dr. Fisher—If we have a case of that kind are we justified in using local treatment, for instance, hot water alone? Is there any danger of assuaging suffering by such treatment as that? Should Dr. Butler do anything whatever, or let the patient lie in bed and say you will be better to-morrow?

Dr. Butler—I gave lots of Sac. lac. and water. It is not my intention to direct discussion from the subject of the paper. Two points there ably presented seemed illustrated by this case, and for that reason I have presented it. First, the mental improvement as a characteristic of remedial action and general improvement. Second, the reappearance of old symptoms as a surety of curative drug action and the necessity for utmost care in the next prescription. I know that the general rule is when old symptoms reappear, *wait*. Do not repeat and do not change your drug.

Dr. W. J. H. Emory—If Dr. Butler's case is disposed of, I would like to say something in the line of the sequelæ of attacks of inflammatory rheumatism. I have a case in point I should like to quote; but, first of all, I would like to state that I have never had any attack of heart trouble following attacks of rheumatism, and have never known of such a case from homœopathic treatment. A case occurred in my practice two or three years ago. The patient had, previously to my treating him, three attacks of inflammatory rheumatism, and all under allopathic treatment. After the last attack he found that his heart was troubling him, and also during the acuteness of the attack he noticed it. He came into my office. He was a stout gentleman, extremely nervous and frightened about his heart, saying he had inflammatory rheumatism two or three years previous, and his heart had been troubling him more or less ever since. I made a stethoscopic examination of the chest and heart, and found a very distinct mitral regurgitant murmur. I did not prescribe for him that evening, and did not have time then, as the next morning I was telephoned for to go and see him, and found he was in for another attack of inflammatory rheumatism—his right wrist was much swelled, and extremely sensitive, and he had got also Chloroform and Laudanum, which he had been applying all night. I at once stopped that, and prescribed for him—Mercurius was the remedy. The aggravation at night, extreme restlessness, and becoming worse on getting warm in bed, and chilliness on moving. I gave him Mercurius$^{2c}$, three doses two hours apart, and that was all the medicine he got, and he progressed favorably every day. It went from the right to the left wrist, and from there to the left ankle, but he suffered very little in comparison with the former sufferings of the previous attacks. This allopathic wrist was the last to get well, and troubled him for two or three weeks. Afterward he came into my office one day, and I examined his heart, and the murmur was gone, and since then he had had no return of the old heart symptoms whatever, and before sailing for Europe he passed an examination for life insurance for $50,000.

Dr. Kent—Dr. Allen's was a very important question—the inference that homœopathic treatment would never permit rheumatism to attack the heart. What is the homœopathic treatment? You may infer that we never make mistakes, but we do sometimes. I will tell you a case where I made a mistake, and wherein I thought I was right beforehand, where I did antidote my medicine and change my plan and remedy, which is quite unusual with me in rheumatism. The case was one that came into the hospital, and it is one of very rare occurrence in practice, and a very peculiar one—a girl of about fourteen years of age, and seemed to be weakly in constitution. When I saw the case her fingers and toes were greatly distended and swollen, and she was so sore that no part of the body could be moved; even her thighs were distended, and her knees and ankles greatly swollen, and could not bear to be touched. I found out that the first evidence she had that she was sick was from the heart feeling bad. She said she had never been strong. While lifting a coal-bucket she felt a krick in her back, and said she stopped, and could not carry the coal upstairs. The pain greatly increased, and extended to all the extremities, and swelling came on. I could not get any history of cold, or taking cold, but found her in this condition. She was *relieved by heat.* There was a great amount of soreness, and intense aching, which was ameliorated by turning in bed. Details of the symptoms I am not able to give, but Rhus seemed to be indicated; she was a restless subject. I gave Rhus, and the rheumatism disappeared from the lower extremities almost by magic in two days. There was cardiac murmur, and the mental symptoms were all violent, and there was increased swelling of the fingers; she had to throw off all the clothes, as she could not tolerate heat. The whole thing was reversed, but accurate symptoms had come on now, and after studying that case, Rhus seemed insufficient—it was not homœopathic to it, yet it was in accordance with the superficial symptoms of the case, and hence in harmony with the paper. Finally, the third day, the rheumatism passing upward with aggravation by heat, amelioration by cold, led me to choose "*Ledum*," which in a few days wiped out the symptoms of the entire attack.

Dr. H. C. Allen—Did that Rhus have any effect on the symptoms?

Dr. Kent—I think they became worse immediately after.

Dr. Allen—Were the symptoms all going up?

Dr. Kent—The rheumatism went upwards.

Dr. Biegler—The case just illustrated by Dr. Kent brings up to my mind a case I have now under treatment. It is a case of a boy, twelve or thirteen, who has been subject to very bad inflammatory attacks. He has had several in his life, all of which I have brought him through myself. This time his history is that he fell off from a bicycle; it was a very high one, and it was his first ride, and he sustained severe concussion. Now, this case is principally one of a constitutionally rheumatic subject; the fall came in as an element of disturbance, and it was not productive of rheumatism, but was an element in the case, like Dr. Kent's case. It was to my mind a case for Rhus, which he received. There was that aversion to cold, restlessness, and the amelioration of symptoms by motion; also, a condition of straining, for which class of symptoms I gave Rhus. The remedy failed, except that, like Dr. Kent's case of rheumatism, it receded from the ankles, where it first showed itself, to the upper extremities. There was also the similar condition, *aversion to heat,* and marked inability to lie down. He was obliged to sit up day and night; it was the only position in which he could remain comfortable. But as the rheumatism had commenced in the ankles and went upwards, we will have taken it in sufficient time, and taking the slow and sure course, I gave him Ledum, which failed entirely to relieve him. These conditions remaining the same, only growing worse, he suffered severely—and, by the way, had one of the *worst organic disturbances of the heart I ever saw him with.* Pulsatilla relieved him, one dose almost immediately; but then, from over-eating and various other causes, of which I know nothing, he had two or three other relapses, and at one time he suffered such excruciating pain in the region of the heart that I looked for other remedies, and found "Kalmia." I gave him a dose, and he was relieved within a wonderfully short time, I may say in almost a few moments, and remained so; but there again came a relapse and the inability to lie down, so that Pulsatilla was again resorted to, with a similar effect, and to such an extent that he is fairly convalescent. With one dose of Pulsatilla he recovered wonderfully well. What I wish to say is that *I believe in homœopathic practice we may get organic affections of the heart from metastasis of rheumatism*, but that we *we can cure them.*

I am satisfied, from the examination I made the day before yesterday of this boy, that it would take a very good ear of a very good diagnostician to discover that that heart had ever been affected. Dr. Schmitt has once or twice seen the boy; he can corroborate my statements as to the condition of his heart. If we unfortunately in any case obtain heart complications from metastasis of rheumatism, we must cure them, because we can do it; and I believe that even in young life, where the heart has been left affected, where nothing has been done except in the old-school fashion, after the disease has become chronic and established; even this we can almost cure, and I have almost wished to say we can cure. I have restored such cases to such an extent that they are now called well, although it might be possible to discern still some trace of valvular disease.

Dr. Sawyer—It seems that Dr. Butler had a Belladonna case. Did Belladonna change that to a Bryonia case? It became clearly a Bryonia case. Now, why? Because this intensely acute case, after standing three days (seventy-two hours) without any change, and not corresponding to the remedy preceding it, why it should be held any longer on that remedy is past my comprehension. The intensely acute condition is not changed in seventy-two hours by the remedy; is not changed by it at all; so, if there had been no mistake in the Bryonia case (*i. e*, that is, in its being a Bryonia case), he was quite right in giving Bryonia.

Dr. Bell—I would like to correct my own impression; he did wait. I think he waited long enough; and certainly in accordance with the laws of homœopathics. I, however, had agreed to the view previously taken. It seems to me it requires greater attention to materia medica on this point brought out in the paper about Bryonia; it teaches you not only the good to do, but how to do it. There are cases where the younger practitioners fail. Of course, it often becomes a question of judgment.

In regard to this particular case, Hahnemann teaches that in a chronic case, when acute symptoms arise, we take a new photograph of the case, and I think it is in accordance with the principle we are speaking about.

Dr. Schmitt—I think we have heard one of the best papers we have heard for a long time; but I never heard such a clear explanation of the appreciation of symptoms as in this paper. This point struck me especially—if the remedy causes any symptoms that have not been indicated, then read up your *Materia Medica* and find out if these symptoms belong to the remedy, and if not it is acting wrong; then select another remedy. Now, very often Hahnemann does not explain as well as Dr. Wesselhœft, and I have been misled in my practice; and when I thought it was a homœopathic aggravation it was the wrong action of the remedy and the patient suffered for two weeks longer—and I thought here is an aggravation, and I should have selected another remedy anyway. If I had known that before I would have selected the right remedy two weeks previously. I want to call your attention to that one point.

Dr. E. A. Ballard—Mr. President, it is just ignorance on the point so well elucidated in that paper that has been mostly the cause of spoiling a great many cases, and it has not been long since I have done that. There is one point, however, I want to speak of in respect to a case I have had recently. A lady passing through the climaxis six months ago for symptoms of long standing and those that were very prominent, I gave a dose of Lachesis, and the case was relieved; then comes in the truth of that paper. Again, in the neighborhood of a month ago she came back to my office, and the same symptoms were prominent. I did not touch the case but gave Sac. lac., and she went along well; and she could not do without these Sac. lac. powders; they always helped her. Afterward these symptoms came up a third time; I gave her nothing and they passed off. I am speaking of this case, however, because I want to show what a "bull" I think I have made; and that paper reminded me of it. About two or three weeks ago she had an attack of tonsillitis, which I did not know of before. She told me she was in the habit of having these attacks frequently. She has been under the ordinary homœopathic treatment, the tonsillitis going along to suppuration almost without exception. This attack showed Mercurius very decidedly; she received a dose, and in a few hours the attack was almost completely annihilated; but she sighs, "I have now the symptoms which I always had with the previous attack, that is," she says, "I want to sleep, but as soon as I sink to sleep I wake up panting for breath, and it seems to me that I had a tremendous hole in my left side, and my life went out through that hole." I did not interfere with that case, but I said, If you cannot sleep to-night and are no better to-morrow (she could not lie down) let me know, and I will endeavour to help you. But I am not interfering with that symptom ; I am giving treatment for sore throat. That symptom caused nothing; two weeks later it had passed away. But here I have records of old Lachesis symptoms—many symptoms which she had showed me, but entirely new—decidedly Lachesis. I gave her a dose 11 mill. I am afraid I have made a "bull" of it. I don't know.

Dr. Bell—Was that a symptom of Mercurius also?

Dr. Ballard—I cannot find it and I did not interfere with the symptoms at the time.

Dr. H. C. Allen—I think you will find it in the *Guiding Symptoms*, under Merc. iodid.

Dr. Ballard—I think that it is interference in these cases that has done a great deal of harm. These symptoms which are entirely new—I mean to say, symptoms which belong to the remedy—we say because of that we have made a wrong selection and must give another remedy. It may answer in chronic cases, but in acute cases we frequently find that after giving a dose, perhaps not an aggravation of the symptoms which we have covered, but an addition of others, which we cover by the other remedy, and we made a mistake in giving it.

Dr. H. C. Allen—Dr. Biegler, I think, has misunderstood me. I have never seen organic lesions of the heart under homœopathic treatment; I meant *permanent*. We can cure the trouble in the heart just as well as anywhere else.

Dr. Long—I feel I have not done my duty in allowing that assertion to go, for I had a patient die from organic heart disease, the result of rheumatism. It was a boy fifteen years of age, whom I treated for two weeks for gastritis, and he got perfectly well. I was away on a week's vacation and on returning found the boy out around, and on Sunday evening he stopped to see me, and at eleven P. M. I was called to see that boy die as I entered the room. He was undoubtedly dropsical ; he was panting for breath and with every motion made, froth and blood came from him, and he gasped his last as I entered the room. Instead of giving the remedy I assisted the undertaker to lay out the boy. On pressing the chest I removed fully a quart of frothy water and blood from the mouth. The boy had been around for two weeks and in my office, and was getting fleshy and had complained of nothing. That boy died of hydrothorax and hydropericardium.

Dr. Sawyer—What remedies were used?

Dr. Long—The only remedy used in the case was Rhus. I did not consider the boy very bad; he seemed comparatively well, and when I returned in February I was told he was well.

Dr. Bell—What is the conclusion of the case?

Dr. J. V. Allen—Dr. Long makes the statement that in laying out the boy after death and squeezing the chest a quantity of bloody, frothy mucus came away. I am not in the undertaking business, but my father is, and I used to accompany him to assist him, and we always squeezed the chest, and we always got bloody, frothy mucus from the stomach, and as a rule there will be this mucus from the mouth in every case.

